# Hemodynamic stress‐induced cardiac remodelling is not modulated by ablation of phosphodiesterase 4D interacting protein

**DOI:** 10.1111/jcmm.17468

**Published:** 2022-07-20

**Authors:** Belal A. Mohamed, Manar Elkenani, Sherok Mobarak, Daniel Marques Rodrigues, Karthika Annamalai, Moritz Schnelle, Michael Bader, Gerd Hasenfuss, Karl Toischer

**Affiliations:** ^1^ Department of Cardiology and Pneumology University Medical Center Göttingen Göttingen Germany; ^2^ DZHK (German Centre for Cardiovascular Research) Göttingen Germany; ^3^ Department of Clinical Chemistry University Medical Center Göttingen Göttingen Germany; ^4^ Max‐Delbrück‐Center for Molecular Medicine (MDC) Berlin‐Buch Germany; ^5^ DZHK (German Centre for Cardiovascular Research) Berlin Germany; ^6^ Charité Universitätsmedizin Berlin Germany

**Keywords:** cardiac remodelling, PKA compartmentalization, pressure overload, volume overload

## Abstract

Adrenergic stimulation in the heart activates the protein kinase A (PKA), which phosphorylates key proteins involved in intracellular Ca^2+^ handling. PKA is held in proximity to its substrates by protein scaffolds, the A kinase anchoring proteins (AKAPs). We have previously identified the transcript of phosphodiesterase 4D interacting protein (*Pde4dip;* also known as myomegalin), one of the sarcomeric AKAPs, as being differentially expressed following hemodynamic overload, a condition inducing hyperadrenergic state in the heart. Here, we addressed whether PDE4DIP is involved in the adverse cardiac remodelling following hemodynamic stress. Homozygous *Pde4dip* knockout (KO) mice, generated by CRISPR‐Cas9 technology, and wild‐type (WT) littermates were exposed to aortocaval shunt (shunt) or transthoracic aortic constriction (TAC) to induce hemodynamic volume overload (VO) or pressure overload (PO), respectively. The mortality, cardiac structure, function and pathological cardiac remodelling were followed up after hemodynamic injuries. The PDE4DIP protein level was markedly downregulated in volume‐overloaded‐ but upregulated in pressure‐overloaded‐WT hearts. Following shunt or TAC, mortality rates were comparably increased in both genotypes. Twelve weeks after shunt or TAC, *Pde4dip*‐KO animals showed a similar degree of cardiac hypertrophy, dilatation and dysfunction as WT mice. Cardiomyocyte hypertrophy, myocardial fibrosis, reactivation of cardiac stress genes and downregulation of ATPase, Ca^2+^ transporting, cardiac muscle, slow twitch 2 transcript did not differ between WT and *Pde4dip‐*KO hearts following shunt or TAC. In summary, despite a differential expression of PDE4DIP protein in remodelled WT hearts, *Pde4dip* deficiency does not modulate adverse cardiac remodelling after hemodynamic VO or PO.

## 1 INTRODUCTION

In response to hemodynamic stress, that is volume overload (VO) and pressure overload (PO), the heart undergoes molecular, structural and functional changes, collectively named cardiac remodelling. This is associated with β‐adrenergic overstimulation,^1^ cyclic adenosine monophosphate (cAMP) synthesis that activates protein kinase A (PKA), which phosphorylates Ca^2+^ regulatory proteins to maintain cardiac output.^2^, ^3^ However, a persistent hyperadrenergic state leads to maladaptive cardiac remodelling, and ultimately to heart failure (HF) and sudden death.^4^

The spatiotemporal tuning of cAMP/PKA signalling is achieved by a group of scaffolding proteins, the A‐kinase anchoring proteins (AKAPs), which held PKA in proximity to its distinct substrates.^5^, ^6^ Additionally, AKAPs associate with phosphatases and phosphodiesterases, thereby providing multistep control of kinase activity to ensure proper subcellular cAMP/PKA compartmentalization.^7^, ^8^ In the heart, several AKAPs are implicated in cardiac pathophysiology.^9^, ^10^, ^11^, ^12^, ^13^, ^14^, ^15^ Hence, regulation of AKAPs activity or expression could be a promising therapeutic strategy for treatment of heart diseases.

Phosphodiesterase 4D interacting protein (PDE4DIP), also referred to as myomegalin, is an AKAP protein that is predominantly expressed in cardiac and skeletal muscle sarcomeres in proximity to the z‐disc and sarcoplasmic reticulum.^16^, ^17^, ^18^, ^19^, ^20^, ^21^ The PDE4DIP anchors PKA to cardiac myosin binding protein‐C (cMyBPC) and cardiac troponin I (cTNI), and therefore facilitates PKA‐mediated phosphorylation of both proteins to augment cardiac contraction under adrenergic stress.^17^, ^22^, ^23^ Taken together, these findings suggest a possible role for PDE4DIP in cAMP/PKA compartmentalization to maintain intracellular Ca^2+^ homeostasis. Therefore, it is not surprising that *Pde4dip* mutations are associated with familial dilated cardiomyopathy and arrhythmia.^24^, ^25^, ^26^ However, the precise role of PDE4DIP in response to pathological stress in vivo remains unknown.

We have previously reported that *Pde4dip* transcript is differentially expressed in the remodelled myocardium upon hemodynamic overload.^27^ Consistently, in the current study, we found that PDE4DIP protein levels are significantly decreased in the mouse heart after VO but increased after PO. However, it remains unknown whether this differential cardiac PDE4DIP expression has a functional role or is only an epiphenomenal event. To explore the role of PDE4DIP in cardiac remodelling, we generated *Pde4dip* knockout (*Pde4dip*‐KO) mice using the CRISPR‐Cas9 technique and exposed them to aortocaval shunt (shunt)‐triggered VO or transthoracic aortic constriction (TAC)‐induced PO. Our data show that PDE4DIP does not seem to be necessary for adverse cardiac remodelling following either VO or PO.

## 2 MATERIALS AND METHODS

### 2.1 Mice

All investigations were approved by the responsible Institutional Review Board (Lower Saxony State Office for Consumer Protection and Food Safety (LAVES), conforms to the *Guide for the Care and Use of Laboratory Animals* published by the US National Institutes of Health (NIH Publication No. 85–23, revised 1985).

### 2.2 Generation of *Pde4dip*‐KO mice with CRISPR/Cas9 system

*Pde4dip*‐KO mice were generated by electroporation of C57Bl/6N zygotes with Cas9 protein and the gRNA GAAGUAUGAAGUCAGCCGGG (AGG), targeting exon 4 of the *Pde4dip* gene. The resulting offspring was genotyped with the primers, forward: 5′‐ TCGCCCACCATGTCTAATGG‐3′, reverse: 5′‐ CCAGATTCAAAGCCCTGTCC‐3′, which bound on flanking sides of the deleted region, followed by sequencing of the PCR fragments. In this study, we analysed male and female wild‐type (WT) and *Pde4dip*‐KO littermates on C57BL/6N genetic background.

### 2.3 Transthoracic aortic constriction

Surgery was done using a minimally invasive technique as described previously.^27^ Briefly, 10‐week‐old WT and *Pde4dip‐*KO mice were anaesthetised using intraperitoneal injections of a mixture of xylazine and ketamine. A 27‐gauge needle was tied against the aorta using a 5–0 non‐absorbable suture. Sham animals underwent the same procedure except banding of the aorta.

### 2.4 Aortocaval shunt

Surgery was done as described previously.^28^ In brief, 10 week‐old mice were anaesthetised using isoflurane, and a mid‐line laparotomy was performed to expose the abdominal aorta and inferior vena cava between the renal arteries and the iliac bifurcation. A 23‐gauge needle was inserted into the exposed aorta at a 45 deg angle and pushed through to the inferior vena cava, creating the shunt. Cyanoacrylate (Pattex) was used to seal the puncture. Sham animals underwent the same procedure except for the creation of the shunt.

### 2.5 Echocardiography

The mice were anaesthetised using 1% isoflurane, and echocardiography was performed using Vevo2100 Imaging Software 3.1.0 (VisualSonics). During this procedure, the body temperature was maintained within physiological range (36°C–37.5°C) using a heating pad, and heart rates were kept consistent between experimental groups at 450–600 b.p.m. Electrocardiogram monitoring was obtained using hind limb electrodes. The left ventricle (LV) geometry and systolic function were assessed by using standard 2D parasternal short axis views in accordance with recommendations where available.^29^ Relative wall thickness (RWT) = (LV anterior wall thickness at diastole + LV posterior wall thickness at diastole) divided by LV end‐diastolic diameter (LVAWTd+LVPWTd/ LVEDD).

The speckle tracking echocardiography was performed as described.^30^ Several tracking points were placed on the endocardial and epicardial border in parasternal long‐axis views. These were used as a guide for border delineation and subsequent frame‐by‐frame tracking throughout the cardiac cycle. The software automatically divides the LV into six segments: two basal, two mid and two apical segments and calculate parameters of deformation (strain, strain rate) and parameters of motion (displacement and velocity), separately for each segment as well as overall mean values. The presented data are the averages from these six different values per heart. The peak longitudinal strain rate during early LV filling, termed as the reverse longitudinal strain rate and the radial diastolic peak velocity were quantified to assess the diastolic function.^31^

### 2.6 Quantitative real‐time polymerase chain reaction

The DNA‐free RNAs were isolated from the left ventricles using RNeasy kit and the RNase‐free DNAse Set (Qiagen), and the RNAs concentration was measured by NANO 2000 (Termal Scientific). The cDNA synthesis was done using the iScript cDNA synthesis kit (Bio‐Rad Laboratories). Real‐time PCR was performed using Bio‐Rad iQ‐Cycler. Transcripts were amplified using SYBR green fluorescent dye and calculated with the delta–delta *C*_t_ method using *Gapdh* as denominator. Primers included for following genes: *Pde4dip* (forward: 5′‐ GAAAACGGTTCCACCTCTCA‐3′; reverse: 5′‐CTTCCTGGGAGTGTCCTCTG‐3′), *Nppa* (forward: 5′‐GGGGGTAGGATTGACAGGAT‐3′; reverse: 5′‐CAGAATCGACTGCCTTTTCC‐3′), *Nppb* (forward: 5′‐ACAAGATAGACCGGATCGGA‐3′; reverse: 5′‐ACCCAGGCAGAGTCAGAAAC‐3′), *Atp2a2* (forward: 5′‐GGGCAAAGTGTATCGACAGG‐3′; reverse: 5′‐TCAGCAGGAACTTTGTCACC‐3′), *Gapdh* (forward: 5′‐ GAGACGGCCGCATCTTCTT‐3′; reverse: 5′‐CAATCTCCACTTTGCCACTGC‐3′).

### 2.7 Immunoblotting

Harvested left ventricle were homogenized in RIPA buffer (Millipore) containing protease and phosphatase inhibitors (Roche Molecular Biochemicals). Twenty μg protein lysates were subjected to sodium dodecyl sulphate polyacrylamide gel electrophoresis. Western blotting was carried out according to standard protocols, using rabbit polyclonal anti‐PDE4DIP (Sigma‐Aldrich), anti‐phospho‐Ser16‐phospholamban (PLN, Badrilla), anti‐phospho‐Ser23/24 Troponin‐I, anti‐Troponin‐I (Cell Signalling Technology) and mouse monoclonal anti‐PLN (Millipore), anti‐GAPDH (Santa Cruz Biotechnology) as primary, and horseradish peroxidase conjugated donkey anti‐rabbit and sheep anti‐mouse immunoglobulin G (Amersham Bioscience) as secondary antibodies. Signals were quantified using enhanced chemiluminescent detection system (Amersham Bioscience) according to the manufacturer's instructions.

### 2.8 Histology

Freshly harvested hearts were fixed in 4% formalin, embedded in paraffin, sectioned (6 μm). Sections were stained with fluorescein‐conjugated wheat germ agglutinin (WGA‐Alexa Fluor 594; Invitrogen) for cross‐sectional area assessment, and with picrosirius red (Abcam) for fibrosis quantification using Image J software (NIH; Bethesda).

### 2.9 Statistical analysis

Statistical analyses were carried out using Prism software version 5.01 (GraphPad Software, Inc) with two‐tailed unpaired Student's *t*‐test or one‐way anova with Bonferroni post‐test correction where appropriate. Kaplan–Meier survival analysis was performed, and a Log‐Rank test was used to determine significance. Data are expressed as mean ± SD or mean ± SEM where appropriate. Differences were considered significant if at least *p* < 0.05.

## 3 RESULTS

### 3.1 PDE4DIP protein is differentially expressed after pathological hemodynamic overload

**FIGURE 1:**
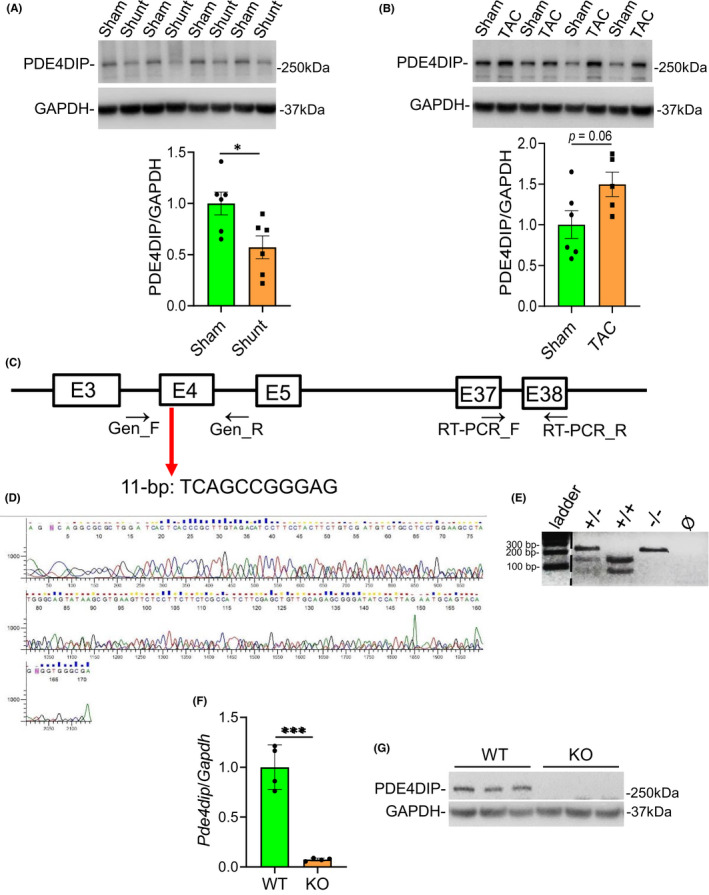
PDE4DIP protein expression is distinctively regulated in remodelled murine hearts. Representative Western blot analysis and quantitative PDE4DIP protein levels in a murine cardiac remodelling induced by aortocaval shunt (A) or transthoracic aortic constriction (TAC) (B). (C) 11 bp DNA deletion in exon 4 of *Pde4dip* gene was introduced via CRISPR/CAS9. The primer pairs, Gen_F, Gen_R and RT‐PCR_F and RT‐PCR_R were used in genotyping PCR and RT‐PCR, respectively. (D) Sequence of the Gen_F/Gen_R PCR product in the heterozygous *Pde4dip* ^*+/−*^ founder. (E) Genotyping by digestion of Gen_F/Gen_R PCR products of WT (*Pde4dip* ^+/+^), heterozygote (*Pde4dip* ^+/−^) and homozygote (*Pde4dip* ^−/−^) mice with restriction enzyme, *NciI*. (F) Quantitative RT‐PCR analysis of the *Pde4dip* mRNA level in WT and *Pde4dip*‐KO hearts. (G) Representative Western blot analysis of PDE4DIP protein level in WT and *Pde4dip*‐KO hearts. Data are mean ± SD, *p < 0.05, ***p < 0.001 versus control, two‐tailed unpaired Student's t test. GAPDH was used as loading control

We assessed PDE4DIP protein level in WT murine remodelled hearts after shunt and TAC, well‐established models of VO‐ and PO‐induced cardiac remodelling, respectively. As shown in Figure 1, PDE4DIP protein levels were differentially expressed in an opposite direction, consistent with our previous microarray data.^27^ Compared with sham control groups, average PDE4DIP protein was significantly reduced by ≈43% in shunt hearts (*p* < 0.05) (Figure 1A) but increased by ≈50% in the TAC hearts (*p* = 0.06) (Figure 1B). These data suggest a potential role for PDE4DIP in myocardial response to hemodynamic stress.

### 3.2 Generation of *Pde4dip*‐KO mice

To get insights into the PDE4DIP function in the heart, we introduced a genomic deletion in mice by CRISPR/Cas9. Genome editing generated a 11 bp deletion located in the coding region of the exon 4 of the *Pde4dip* gene (Figure 1C), resulting in a frameshift mutation, thereby disrupting the reading frame of *Pde4dip*. Heterozygous *Pde4dip* ^+/−^ founders were identified by Sanger sequencing of PCR products covering the edited region (Figure 1D). The heterozygous mice from F1 generation were intercrossed and the genotyping of the offspring was performed by digestion of the PCR products with the restriction enzymes, *NciI* or *HpaII*; both restriction sites are absent in the KO allele. Thus, the WT allele (210 bp) produces two bands of 130 and 80 bp with either of the two enzymes, whereas the KO allele is not cut and therefore gives a 210 bp PCR fragment (Figure 1E). Quantitative RT‐PCR analysis of *Pde4dip*‐KO heart showed a very low *Pde4dip* transcript level, probably due to unstable *Pde4dip* mRNAs with the frameshift deletion (Figure 1F). Western blot analysis clearly showed no detectable PDE4DIP protein in the *Pde4dip*‐KO mice (Figure 1G).

### 3.3 No appreciable structural or functional changes in *Pde4dip*‐KO mice at baseline

**FIGURE 2:**
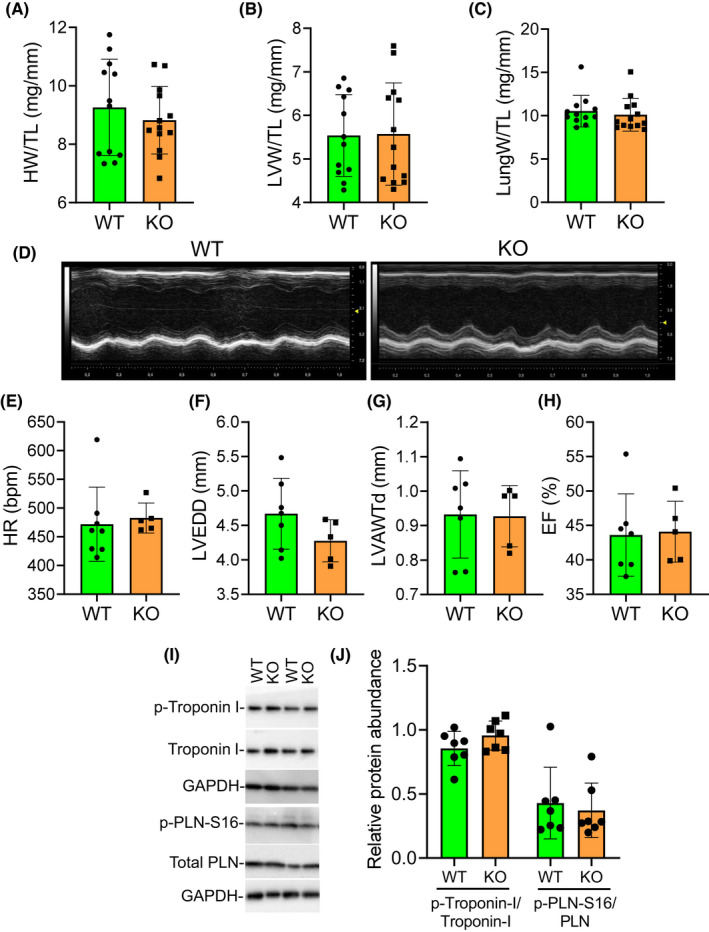
Cardiac structure and function are not different in 12‐month‐old WT and *Pde4dip‐*KO animals. (A–C) Heart weight, left ventricle weight and lung weight normalized to tibia length (HW/TL, LVW/TL, LungW/TL) in *Pde4dip*‐KO and WT mice. (D) Representative echocardiographic M‐mode images from WT and *Pde4dip*‐KO hearts. (E–H) Structural and functional Echo parameters. (I and J) Representative Western blots of Ca ^2+^ regulatory proteins from 3‐month‐old WT and *Pde4dip*‐KO mice (I) and densitometry analysis (J). Data are mean ± SD, two‐tailed unpaired Student's *t* test. HR, heart rate; LVEDD, left ventricular end‐diastolic diameter; LVAWTd, left ventricle anterior wall thickness at diastole; EF, ejection fraction

The *Pde4dip*‐KO mice were born at expected Mendelian ratios (Table S1), and were viable, fertile and exhibited normal life spans. At baseline, heart weight (HW), LV weight (LVW) and lung weight (LungW) to tibia length (TL) ratios were not significantly different between WT and *Pde4dip‐*KO mice (Figure 2A–C). The cardiac geometry and function in *Pde4dip*‐KO mice were similar to that in WT mice up to at least 12 months of age, as determined by echocardiography (Figure 2D–H and Table S2). Compartmentalization of the cAMP‐dependent PKA by AKAPs facilitates local protein phosphorylation at distinct subcellular localization. To test whether PDE4DIP deletion would impair local PKA activity, we measured phosphorylation of key PKA‐dependent Ca^2+^ regulatory proteins; troponin‐I at Ser23/24 and PLN at Ser16. Indeed, we could not detect any differential changes in the phosphorylation pattern of any of these PKA‐targeted proteins between WT and *Pde4dip*‐KO hearts (Figure 2I,J). Thus, *Pde4dip* deletion does not affect basal cardiac structure and function.

### 3.4 Survival, cardiac structure and function are comparable between WT and *Pde4dip*‐KO after shunt

**FIGURE 3:**
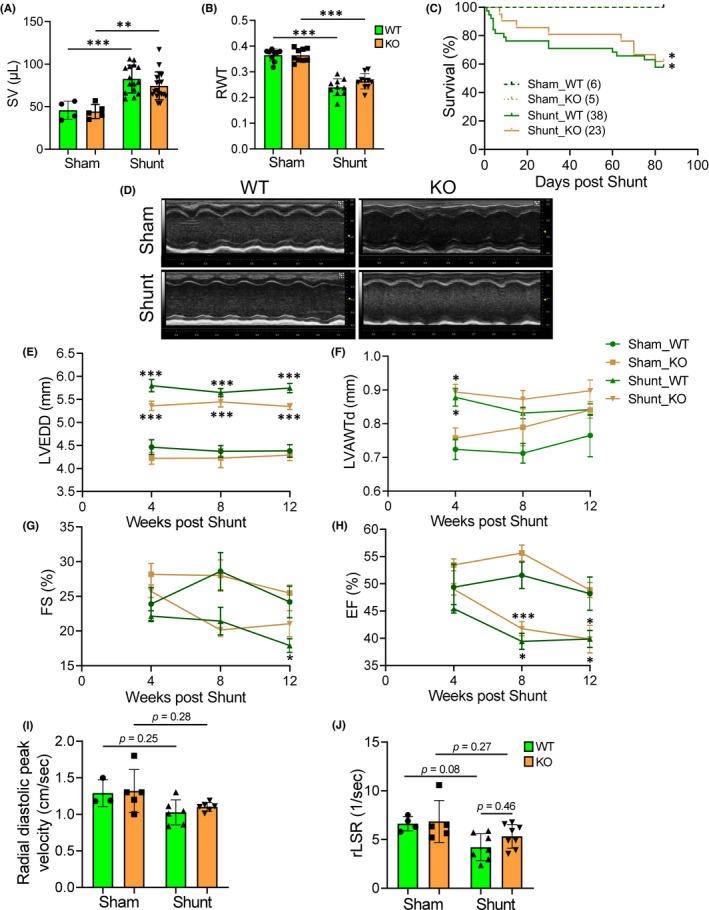
Similar adverse cardiac remodelling in WT and *Pde4dip‐*KO mice after shunt. (A and B) Stroke volume (A) and relative wall thickness (RWT) in WT and *Pde4dip‐*KO mice at 1 weeks post‐shunt (B). (C) Kaplan–Meier survival curves, log‐rank test. (D) Echocardiographic M‐mode representative images at 12 weeks after shunt. (E‐H) LV end‐diastolic diameter, LVEDD (E); LV anterior wall thickness at diastole, LVAWTd (F); fractional shortening, FS (G) and ejection fraction, EF (H). (I and J) Radial diastolic peak velocity (I) and average reverse longitudinal strain rate (rLSR) (J), measured by speckle tracking echocardiography. Data are mean ± SD, *p < 0.05, **p < 0.01, ***p < 0.001 versus corresponding sham, one‐way anova with Bonferroni post‐test

We subjected WT and *Pde4dip‐*KO littermates to chronic VO by shunt. The stroke volume (SV) was significantly, but similarly, increased at 1‐week post‐shunt in both genotypes, indicating comparable VO (Figure 3A). The relative wall thickness (RWT) was significantly, but comparably, lower at 1‐week post‐shunt (Figure 3B), indicating similar eccentric hypertrophy. The mortality rates up to 12 weeks after shunt did not differ between *Pde4dip*‐KO and WT mice (Figure 3C).

Echocardiographic analysis revealed that both WT and *Pde4dip‐*KO mice exhibited early myocardial dilatation, but minimally increased wall thickness after shunt (Figure 3D–F). Fractional shortening (FS) and ejection fraction (EF) progressively but similarly decreased in both genotypes after shunt (Figure 3G,H and Table 1). The global longitudinal systolic strain rate (systolic parameter), radial diastolic peak velocity and peak reverse longitudinal strain rate (diastolic parameters) were mildly but comparably deteriorated in both genotypes at 12 weeks after shunt (Figure 3I,J; Table 1).

**TABLE 1:** Echocardiographic and morphometric analyses of WT and *Pde4dip‐*KO mice at 12 weeks after shunt

	sham‐WT	sham‐KO	shunt‐WT	shunt‐KO
*n*	6	6	18	11
Echocardiography				
HR (bpm)	482 ± 16.1	490.17 ± 12.6	475.33 ± 13.6	485.45 ± 16.5
LVESD (mm)	3.33 ± 0.18	3.20 ± 0.13	4.72 ± 0.10***	4.23 ± 0.14***^#^
LVEDD (mm)	4.38 ± 0.14	4.29 ± 0.12	5.75 ± 0.10***	5.40 ± 0.07***
AWThd (mm)	0.77 ± 0.06	0.84 ± 0.02	0.84 ± 0.02	0.90 ± 0.03
PWThd (mm)	0.58 ± 0.01	0.70 ± 0.04^#^	0.69 ± 0.02*	0.73 ± 0.03
SV (μl)	43.44 ± 2.26	43.12 ± 2.68	72.68 ± 4.13***	61.18 ± 3.25*
EF (%)	48.20 ± 3.03	48.86 ± 1.40	39.89 ± 1.57*	40.42 ± 2.56*
FS (%)	24.19 ± 2.27	25.48 ± 1.15	17.91 ± 0.98*	21.65 ± 1.92
CO (ml/min)	18.57 ± 1.23	19.77 ± 1.19	32.61 ± 2.44**	26.39 ± 2.04
LV Mass (mg)	105.30 ± 8.3	114.94 ± 13.1	170.18 ± 8.4***	171.89 ± 12.4***
Rel. wall thickness	0.31 ± 0.01	0.32 ± 0.01	0.27 ± 0.01*	0.30 ± 0.01*
GLS	−16.12 ± 0.52	−14.27 ± 0.29	−12.20 ± 1.20	−11.19 ± 1.38
Radial peak velocity (d)	1.29 ± 0.105	1.27 ± 0.18	1.08 ± 0.08	1.15 ± 0.04
rLSR	6.63 ± 0.37	6.84 ± 0.96	4.21 ± 0.52	5.33 ± 0.43

*n*	5	5	6	6
Morphometry				
BW/TL (mg/mm)	1.23 ± 0.03	1.27 ± 0.02	1.52 ± 0.01*	1.49 ± 0.03*
LVW/TL (mg/mm)	4.74 ± 0.25	4.83 ± 0.14	9.78 ± 0.64**	10.26 ± 0.90**
LungW/TL (mg/mm)	7.74 ± 0.16	7.64 ± 0.14	9.87 ± 0.36*	9.17 ± 0.26*
Fulton index	0.22 ± 0.01	0.21 ± 0.01	0.29 ± 0.02*	0.31 ± 0.02*
LiverW/TL (mg/mm)	54.84 ± 0.54	58.00 ± 0.71	67.30 ± 1.85*	70.41 ± 2.14*
SpleenW/TL (mg/mm)	4.76 ± 0.31	4.95 ± 0.57	6.92 ± 0.39*	6.43 ± 0.35*

*Note*: Data are mean ± SEM. *n* indicates number of mice.

Abbreviations: AWThd, anterior wall thickness at diastole; bpm, beats per minute; BW/TL, body weight to tibia length ratio; CO, cardiac output; EF, ejection fraction; FS, fractional shortening; Fulton index, right ventricular weight standardized by the left ventricular and interventricular septum weight (RV/[LV + S]); GLS, Global longitudinal systolic strain rate; HR, heart rate; KO, knock out; LVEDD, left ventricular end‐diastolic diameter; LVESD, left ventricular end‐systolic diameter; LV Mass, left ventricular mass; LVW/TL, left ventricle weight to tibia length ratio; LiverW/TL, liver weight to tibia length ratio; LungW/TL, lung weight to tibia length ratio; PWThd, posterior wall thickness at diastole; Radial peak velocity (d), Radial peak velocity at diastole; rLSR, peak reverse longitudinal strain rate; spleenW/TL, spleen weight to tibia length ratio; SV, stroke volume; WT, wild‐type.

*p < 0.05, **p < 0.01, ***p < 0.001 vs. corresponding sham; ^*#*^ *p* < 0.05 vs. corresponding WT, one‐way anova with Bonferroni post‐test correction.

Consistent with biventricular VO induced by shunt, morphometric analysis at 12 weeks post‐shunt revealed significant LV hypertrophy, as evidenced by increased ratio of the LVW to TL, and marked right ventricle (RV) hypertrophy, as judged by increased Fulton index, was no difference between WT and *Pde4dip*‐KO hearts (Table 1). LungW was comparably increased after shunt, suggesting congestive HF in both genotypes (Table 1). Pathologically elevated central venous pressure, characterized by hepatic congestion (increased liverW‐to‐TL), splenic congestion (increased spleenW‐to‐TL) and peripheral oedema (increased total bodyW‐to‐TL), was evident after shunt, suggesting similar RV dysfunction in both genotypes (Table 1).

**FIGURE 4:**
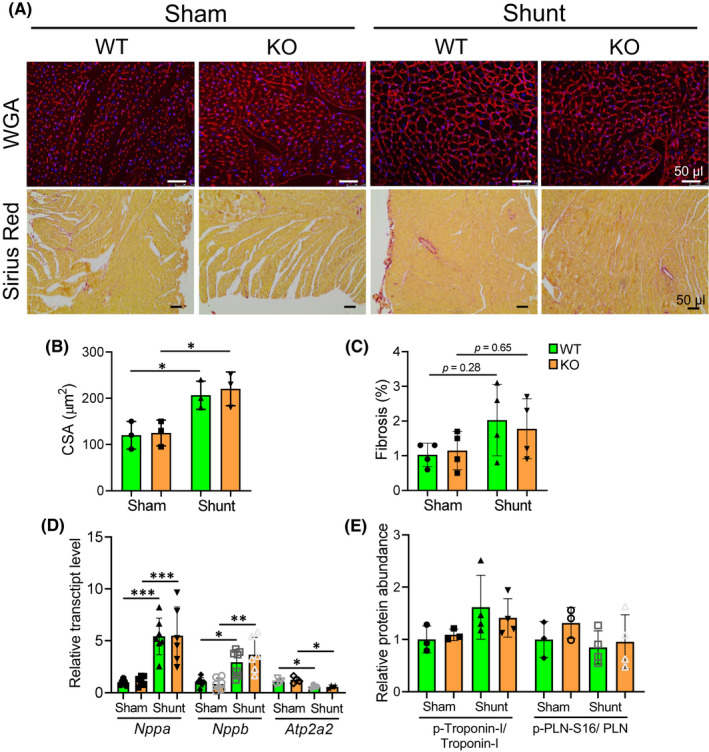
Indistinguishable pathological myocardial remodelling and intracellular Ca^2+^ regulatory proteins level in WT and *Pde4dip‐*KO hearts at 12 weeks after shunt. (A) Representative transverse sections from WT and *Pde4dip*‐KO hearts stained with wheat germ agglutinin (WGA, upper panels) and picrosirius red (lower panels). (B and C) Cardiomyocyte cross sectional area (CSA) (B) and fibrosis (% area) (C). (D) Relative mRNA level of *Nppa*, *Nppb* and *Atp2a2* in WT and *Pde4dip‐*KO heart*s*. (E) Densitometry analysis of Ca^2+^ regulatory protein expression. Data are mean ± SD, *p < 0.05, **p < 0.01, ***p < 0.001 versus corresponding sham, one‐way anova with Bonferroni post‐test

Histological analysis revealed no differences between WT and *Pde4dip*‐KO hearts at 12 weeks after shunt (Figure 4A–C). Cardiomyocyte hypertrophy did not differ between shunt‐operated WT and *Pde4dip*‐KO hearts, as evidenced by similar increased cross surface areas (Figure 4A,B). Shunt hearts showed a tendency towards increased myocardial fibrosis, with no difference between genotypes (Figure 4A,C). Molecular analysis showed similar upregulation of cardiac stress transcripts, natriuretic peptide type A (*Nppa*) and natriuretic peptide type B (*Nppb*) and downregulation of ATPase, Ca^2+^ transporting, cardiac muscle, slow twitch 2 (*Atp2a2*) in WT and *Pde4dip*‐KO hearts after shunt (Figure 4D). The PKA‐dependent phosphorylation of troponin‐I and PLN did not differ between WT and *Pde4dip*‐KO hearts in sham or shunt groups (Figure 4E and Figure S1).

Overall, our data illustrate that both WT and *Pde4dip‐*KO mice experienced comparable adverse cardiac remodelling after shunt.

### 3.5 Similar pathological cardiac remodelling in WT and *Pde4dip‐*KO mice following TAC

**FIGURE 5:**
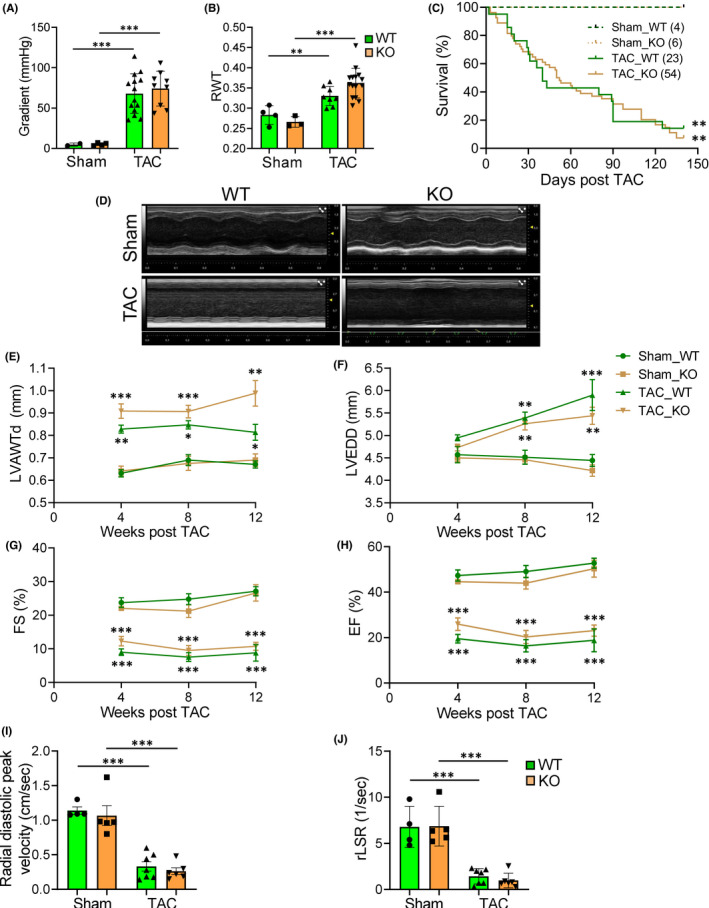
Equal deterioration of cardiac structure and function in WT and *Pde4dip‐*KO mice after TAC. (A) Trans‐stenotic systolic pressure gradient measurement at 2 days after TAC. (B) Relative wall thickness (RWT) at 1 week post‐TAC. (C) Kaplan–Meier survival curves, log‐rank test. (D) Echocardiographic M‐mode representative images at 12 weeks after TAC. (E‐H) LV anterior wall thickness at diastole, LVAWTd (E); LV end‐diastolic diameter, LVEDD (F); fractional shortening, FS (G) and ejection fraction, EF (H). (I and J) Radial diastolic peak velocity (I) and average reverse longitudinal strain rate (rLSR) (J), measured by speckle tracking echocardiography. Data are mean ± SD, *p < 0.05, **p < 0.01, ***p < 0.001 versus corresponding sham, one‐way anova with Bonferroni post‐test

Although both are hemodynamic stresses, PO and VO induce different functional and molecular adaptations in cardiac hypertrophy, causing morphologically distinct types of cardiac remodelling.^27^, ^28^ We therefore extended our study and exposed WT and *Pde4dip‐*KO littermates to chronic PO by TAC. The pressure gradient across the aortic constriction was similarly increased in WT and *Pde4dip‐*KO mice after TAC (Figure 5A), indicating an equal degree of PO and comparable wall stress. Both WT and *Pde4dip*‐KO hearts responded to TAC with an early (1‐week post‐TAC) and significant increased RWT, indicative of concentric hypertrophy (Figure 5B). Within 20 weeks of PO, both genotypes exhibited similar mortality patterns (Figure 5C).

Cardiac geometry and function were evaluated using serial echocardiography (Figure 5D). At 4 weeks after surgery, both TAC mice exhibited massively increased wall thickness, but minimal chamber dilatation versus corresponding sham mice (Figure 5E,F). Beyond this time frame, both TAC mice similarly experienced progressive LV dilatation with further but minimal increase in the wall thickness (Figure 5E,F). Cardiac structure, FS and EF declined similarly in WT and *Pde4dip‐*KO mice after TAC (Figure 5G,H and Table 2). The global longitudinal systolic strain rate was significantly reduced in both genotypes at 12 weeks post‐TAC, confirming systolic impairment (Table 2). Furthermore, the diastolic parameters, radial diastolic peak velocity and peak reverse longitudinal strain rate, were markedly decreased in both genotypes at 12 weeks post‐TAC, indicating a comparable diastolic dysfunction (Figure 5I,J and Table 2).

**TABLE 2:** Echocardiographic and morphometric parameters of WT and *Pde4dip‐*KO mice at 12 weeks after TAC

	sham‐WT	sham‐KO	TAC‐WT	TAC‐KO
*n*	5	6	9	12
Echocardiography				
HR (bpm)	493.56 ± 14.9	508.83 ± 17.8	498.75 ± 36.67	528.42 ± 11.59
LVESD (mm)	3.25 ± 0.15	3.11 ± 0.17	5.40 ± 0.43***	4.87 ± 0.23***
LVEDD (mm)	4.44 ± 0.13	4.22 ± 0.13	5.90 ± 0.35***	5.44 ± 0.19**
AWThd (mm)	0.67 ± 0.02	0.69 ± 0.03	0.81 ± 0.04*	0.99 ± 0.06**
PWThd (mm)	0.67 ± 0.01	0.71 ± 0.03	0.86 ± 0.03*	0.95 ± 0.06**
SV (μl)	46.92 ± 1.88	40.90 ± 2.70	29.52 ± 6.53**	30.84 ± 2.28
EF (%)	52.79 ± 2.23	50.31 ± 3.72	18.77 ± 5.00***	23.05 ± 2.46***
FS (%)	27.10 ± 1.38	26.62 ± 2.44	8.82 ± 2.46***	10.76 ± 1.19***
CO (mL/min)	21.19 ± 0.92	19.62 ± 0.92	13.82 ± 2.79*	15.74 ± 1.27*
LV Mass (mg)	113.09 ± 16.1	122.03 ± 6.4	236.09 ± 17.4***	259.82 ± 34.1***
Rel. wall thickness	0.30 ± 0.01	0.32 ± 0.02	0.29 ± 0.02	0.36 ± 0.03
GLS	−15.81 ± 0.40	−14.27 ± 0.3	−2.90 ± 0.87***	−1.93 ± 0.62***
Radial peak velocity (d)	1.14 ± 0.05	1.07 ± 0.14	0.33 ± 0.07***	0.26 ± 0.05***
rLSR	6.78 ± 1.12	6.85 ± 0.96	1.42 ± 0.31***	0.98 ± 0.32***

*n*	5	5	7	7
Morphometry				
BW/TL (mg/mm)	1.25 ± 0.01	1.33 ± 0.03	1.23 ± 0.06	1.22 ± 0.04
LVW/TL (mg/mm)	4.73 ± 0.05	4.79 ± 0.14	10.05 ± 0.57**	9.98 ± 0.39**
LungW/TL (mg/mm)	10.76 ± 0.74	9.18 ± 0.66	24.99 ± 1.89**	21.22 ± 1.10**
Fulton index	0.20 ± 0.01	0.20 ± 0.01	0.17 ± 0.02	0.19 ± 0.01
LiverW/TL (mg/mm)	59.44 ± 4.32	65.51 ± 2.95	66.31 ± 4.31	59.17 ± 3.08
SpleenW/TL (mg/mm)	4.28 ± 0.24	4.14 ± 0.22	4.73 ± 0.21	4.83 ± 0.50

*Note*: Data are mean ± SEM. *n* indicates number of mice.

Abbreviations: AWThd, anterior wall thickness at diastole; bpm, beats per minute; BW/TL, body weight to tibia length ratio; CO, cardiac output; EF, ejection fraction; FS, fractional shortening; Fulton index, right ventricular weight standardized by the left ventricular and interventricular septum weight (RV/[LV + S]); GLS, Global longitudinal systolic strain rate; HR, heart rate; KO, knock out; LVEDD, left ventricular end‐diastolic diameter; LVESD, left ventricular end‐systolic diameter; LV Mass, left ventricular mass; LVW/TL, left ventricular weight to tibia length ratio; LiverW/TL, liver weight to tibia length ratio; LungW/TL, lung weight to tibia length ratio; PWThd, posterior wall thickness at diastole; Radial peak velocity (d), Radial peak velocity at diastole; rLSR, peak reverse longitudinal strain rate; spleenW/TL, spleen weight to tibia length ratio; SV, stroke volume; WT, wild‐type.

*p < 0.05, **p < 0.01, ***p < 0.001 vs. corresponding sham, one‐way anova with Bonferroni post‐test correction.

**FIGURE 6:**
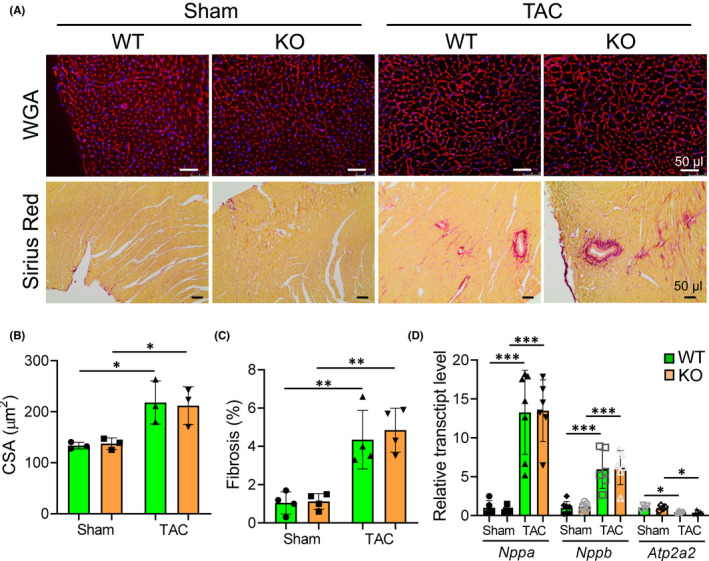
Comparable cardiomyocyte hypertrophy, cardiac fibrosis and fetal genes expression in WT and *Pde4dip‐*KO mice at 12 weeks after TAC. (A) Representative transverse sections from WT and *Pde4dip‐*KO hearts stained with wheat germ agglutinin (WGA, upper panels) and picrosirius red (lower panels). (B and C) Quantification of cardiomyocyte cross sectional area (CSA) (B), and fibrosis (% area) (C). (D) Relative transcript levels of *Nppa*, *Nppb* and *Atp2a2*. Data are mean ± SD, *p < 0.05, **p < 0.01, ***p < 0.001 versus corresponding sham, one‐way anova with Bonferroni post‐test

Quantification of cardiomyocyte hypertrophy revealed no differences in myocyte surface areas in TAC‐operated WT and *Pde4dip*‐KO mice (Figure 6A,B). Picrosirius red staining showed similar myocardial fibrosis between WT and *Pde4dip*‐KO hearts after TAC (Figure 6A,C). TAC‐induced cardiac hypertrophy was mainly due to LV hypertrophy, as reflected by marked increased in LVW‐to‐TL ratio (Table 2). LungW‐to‐TL ratio was markedly increased in both genotypes after TAC (Table 2). WT and *Pde4dip‐*KO animals showed a comparable upregulation of *Nppa* and *Nppb* and downregulation of *Atp2a2* transcripts post‐TAC (Figure 6D).

Taken together, our data show that both WT and *Pde4dip‐*KO mice experienced equal cardiac dysfunction with signs of manifested HF after TAC.

## 4 DISCUSSION

PDE4DIP interacts with a cAMP‐related PDE4D and PKA and recruits cMyBPC and cTNI at the sarcomere. Its sarcomeric localization is therefore compatible with a mechanism that would augment β‐adrenergic‐mediated phosphorylation of cMyBPC and cTnI to enhance cardiac contraction upon adrenergic stimulation.^16^, ^17^, ^22^, ^23^ We therefore propose that PDE4DIP, beside its structural role as scaffold, could be functionally involved in maintenance of intracellular Ca^2+^ homeostasis within cardiomyocytes. In this study, we tested the role of PDE4DIP during hemodynamic PO and VO using *Pde4dip*‐KO mice. The *Pde4dip*‐KO mice were indistinguishable from WT littermates at baseline, and *Pde4dip* ablation did not alter the adverse cardiac remodelling upon 12 weeks of pathological hemodynamic stress.

Consistent with our previous gene expression data,^27^ we here observed that PDE4DIP protein level is differentially regulated by distinct stimuli; PDE4DIP protein level was markedly downregulated in volume overloaded‐ but upregulated in pressure overloaded‐hearts. The discrepant expression of PDE4DIP between VO and PO might be due to the different stimuli elicited by VO (principally increasing diastolic wall stress) and PO (predominantly increasing wall thickness). At present, the signalling mechanism that regulate PDE4DIP expression and processing following hemodynamic stress remain unclear, which warrants further investigation.

Theoretically, PDE4DIP protein levels could be differentially regulated in remodelled hearts because it is involved in hypertrophic response. Alternatively, differential expression could be a counter‐regulatory mechanism. Thus, *Pde4dip* deletion should reveal the function of PDE4DIP and lead to either exaggerated or attenuated hypertrophic phenotypes. However, *Pde4dip*‐KO mice did not show any alteration in cardiac structure and function under baseline conditions or modulates adverse remodelling following shunt or TAC. Thus, our data does not support either possibility. A third postulation might be that *Pde4dip* deletion could be compensated by other redundant AKAPs with overlapping functions or subcellular localization. In the heart, more than 30 AKAPs are expressed.^32^ For example, it was previously reported that AKAP7d tethers PKA to the sarcoplasmic reticulum Ca^2+^ pump to regulate Ca^2+^ reuptake.^11^ However, Ca^2+^ cycling in cardiomyocytes was not affected by genetic ablation of AKAP 15/18.^33^ This genetic robustness provides a versatile means to respond to the continually changing stimuli within myocytes. Future studies are therefore needed to address this hypothesis.

We showed here that CRISPR‐mediated targeted deletion of *Pde4dip* does not alter the adverse cardiac remodelling upon pathological hemodynamic stress. We therefore conclude that murine PDE4DIP is not required for cardiac hypertrophy, fibrosis or contractile dysfunction, at least in response to 12 weeks of hemodynamic challenges. It is likely that our current study was not continued long enough to observe any potential delayed effect of *Pde4dip* deletion in end‐stage HF. A full body PDE4DIP ablation might have also some limitations. Deletion of *Pde4dip* during whole life span could affect embryonic development that might potentially influence the extent of cardiac remodelling following hemodynamic stress. Moreover, the global deletion of *Pde4dip* occurs in all cell types and possible unexplored functions of PDE4DIP might therefore influence the outcome.

In summary, although pathological hemodynamic overload markedly alters PDE4DIP protein levels in WT hearts, there was no difference in adverse cardiac remodelling between WT and *Pde4dip‐*KO mice after exposure to VO or PO. Thus, our results do not support a major role of PDE4DIP on myocardial structure and function in healthy heart or following hemodynamic stress.

## AUTHOR CONTRIBUTIONS

**Belal Mohamed:** Conceptualization (equal); data curation (equal); formal analysis (equal); investigation (equal); methodology (equal); project administration (equal); supervision (equal); writing – original draft (equal); writing – review and editing (equal). **Manar El kenani:** Data curation (equal); formal analysis (equal); investigation (equal); methodology (equal). **Sherok mobarak:** Investigation (equal); methodology (equal). **Daniel marquesrodrigues:** Methodology (equal). **karthika annamalai:** Investigation (equal); methodology (equal). **moritz schnelle:** Investigation (equal); methodology (equal). **Michael Bader:** Funding acquisition (equal); investigation (equal); methodology (equal). **Gerd Hasenfuss:** Conceptualization (equal); funding acquisition (equal). **Karl Toischer:** Conceptualization (equal); project administration (equal); resources (lead).

## CONFLICT OF INTEREST

The authors confirm that there are no conflicts of interest.

## Supporting information


Appendix S1

## Data Availability

The data that supports the findings of this study are available in the supplementary material of this article.
