# Supplementary material for: Hemodynamic stress‐induced cardiac remodelling is not modulated by ablation of phosphodiesterase 4D interacting protein
Source: J Cell Mol Med. 2022 Jul 20;26(16):4440–52. doi: 10.1111/jcmm.17468 (PMC9357604; doi:10.1111/jcmm.17468)

**Supporting Information**

**Figure S1.** Representative Western blots of Ca ^2+^ regulatory proteins from 12 weeks sham and shunt-operated WT and *Pde4dip*-KO mice.

**
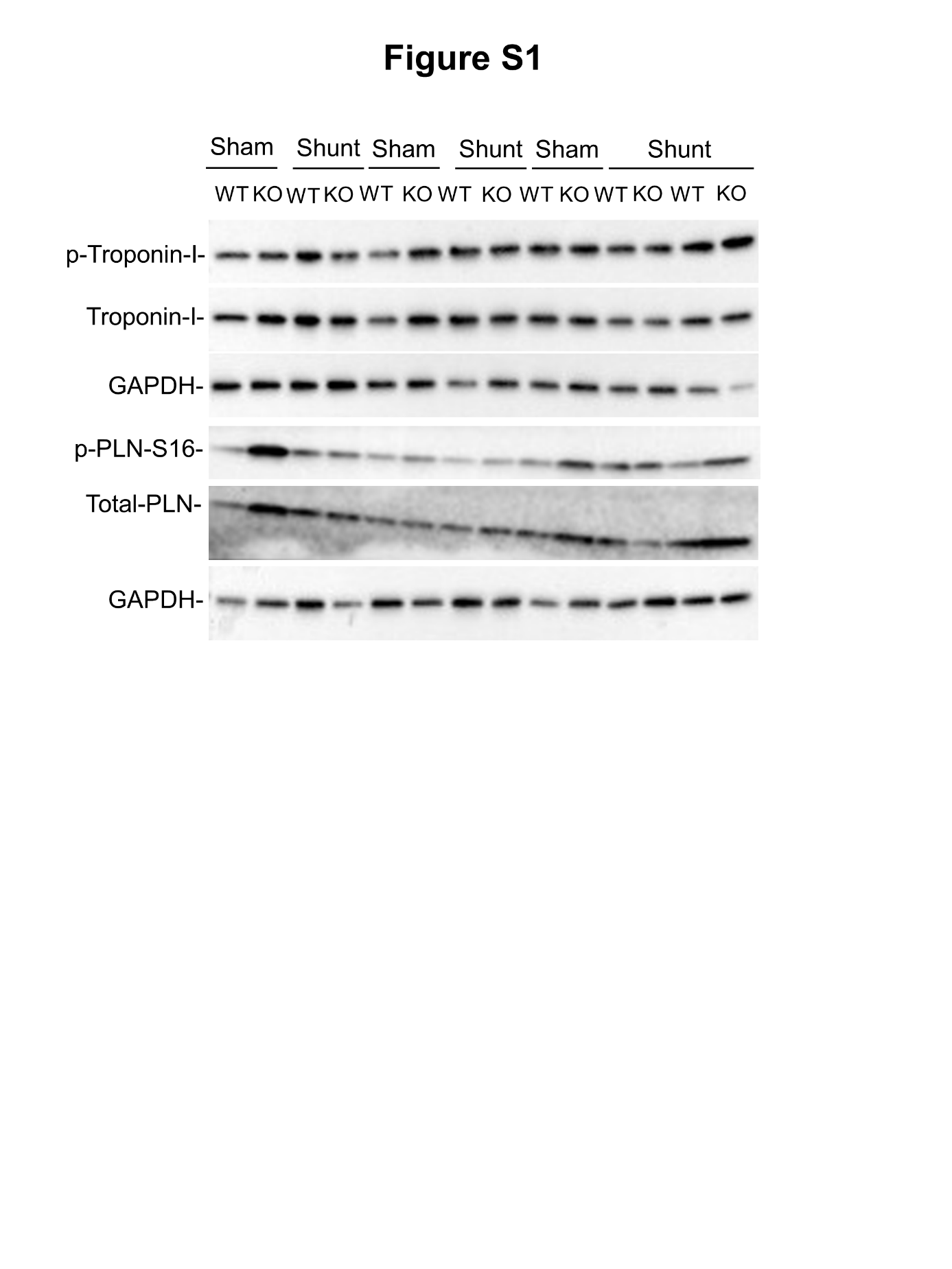
**

**Table S1.** *Pde4dip*-KO mice were born at expected Mendelian ratios

| *Pde4dip* Genotype (112 mice) | Predicted | Observed |
| --- | --- | --- |
| *Pde4dip*^+/+^ | 28 (25%) | 30 (27%) |
| *Pde4dip*^+/-^ | 56 (50%) | 57 (51%) |
| *Pde4dip*^-/-^ | 28 (25%) | 25 (22%) |

**Table S2.** Echocardiography of 12 month-old *Pde4dip*-KO and WT mice

|  | *Pde4dip*^+/+^ (7) | *Pde4dip*^-/-^ (6) |
| --- | --- | --- |
| BW (g) | 29.86± 1.13 | 28.8± 0.68 |
| HR (bpm) | 480.14± 24.67 | 482.8± 11.68 |
| LVESD (mm) | 3.75± 0.18 | 3.23± 0.08^*^ |
| LVEDD (mm) | 4.67± 0.19 | 4.28± 0.14 |
| AWThd (mm) | 0.93± 0.05 | 0.93± 0.04 |
| PWThd (mm) | 0.76± 0.03 | 0.69± 0.04 |
| SV (µl) | 52.40± 5.32 | 37.46± 4.58 |
| EF (%) | 43.61± 2.26 | 44.09± 1.973 |
| FAS (%) | 35.91± 2.30 | 37.98± 1.95 |
| CO (mL/min) | 25.19± 2.89 | 18.08± 2.21 |
| LV Mass (mg) | 135.50± 14.36 | 111.75± 7.58 |
| Rel. wall thickness | 0.36± 0.02 | 0.38± 0.01 |

bpm, beats per minute; AWThd, anterior wall thickness at diastole; BW, body weight; CO, cardiac output; EF, ejection fraction; FAS, fractional area shortening; HR, heart rate; LV Mass, left ventricular mass; LVEDD, left ventricular end-diastolic diameter; LVESD, left ventricular end-systolic diameter; PWThd, posterior wall thickness at diastole; SV, stroke volume; WT, wild-type. *^*^P* < 0.05 vs. WT, two-tailed unpaired Student's *t*-test. Data are mean ± SEM. Numbers within parentheses indicate mice.


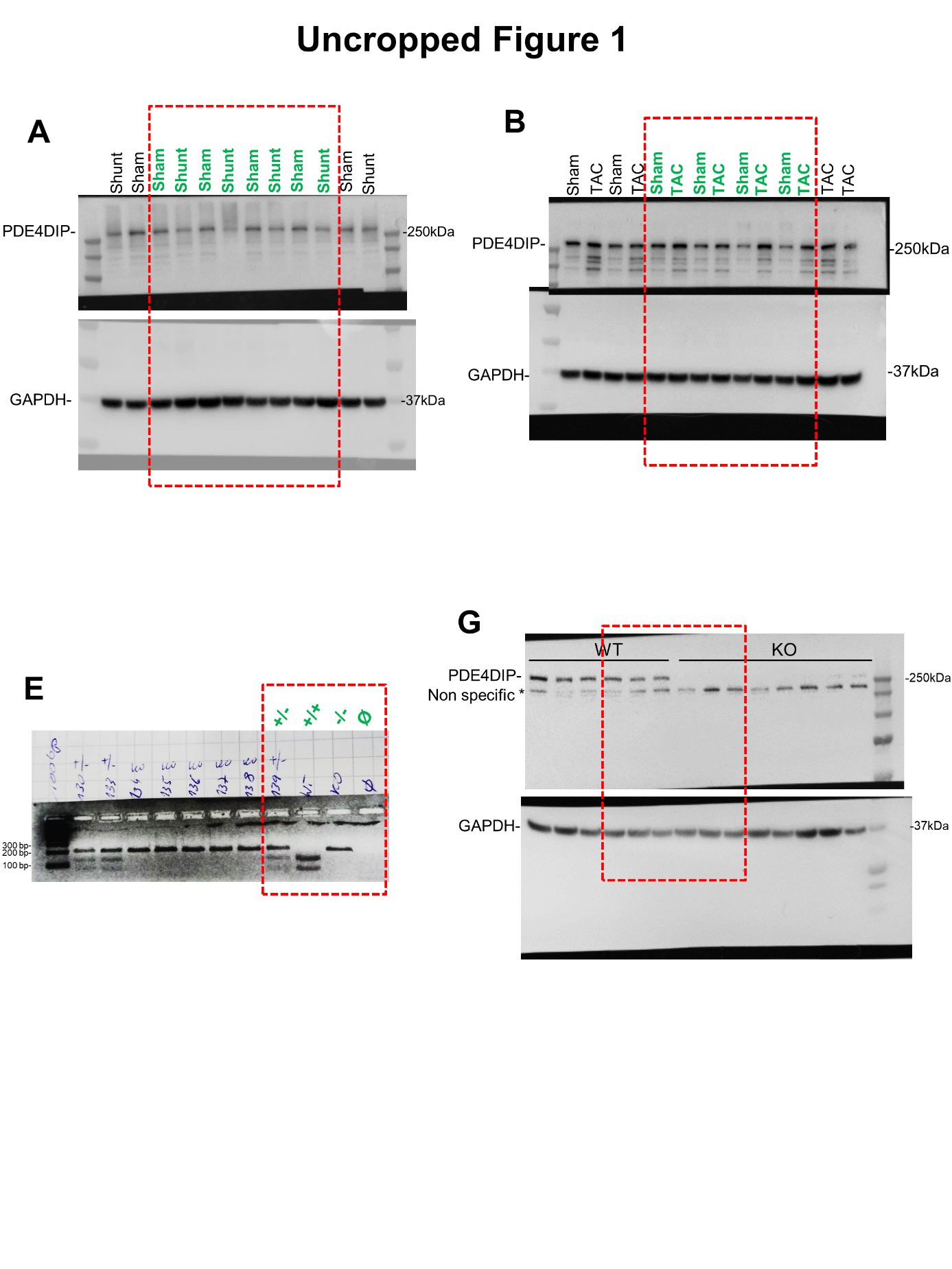


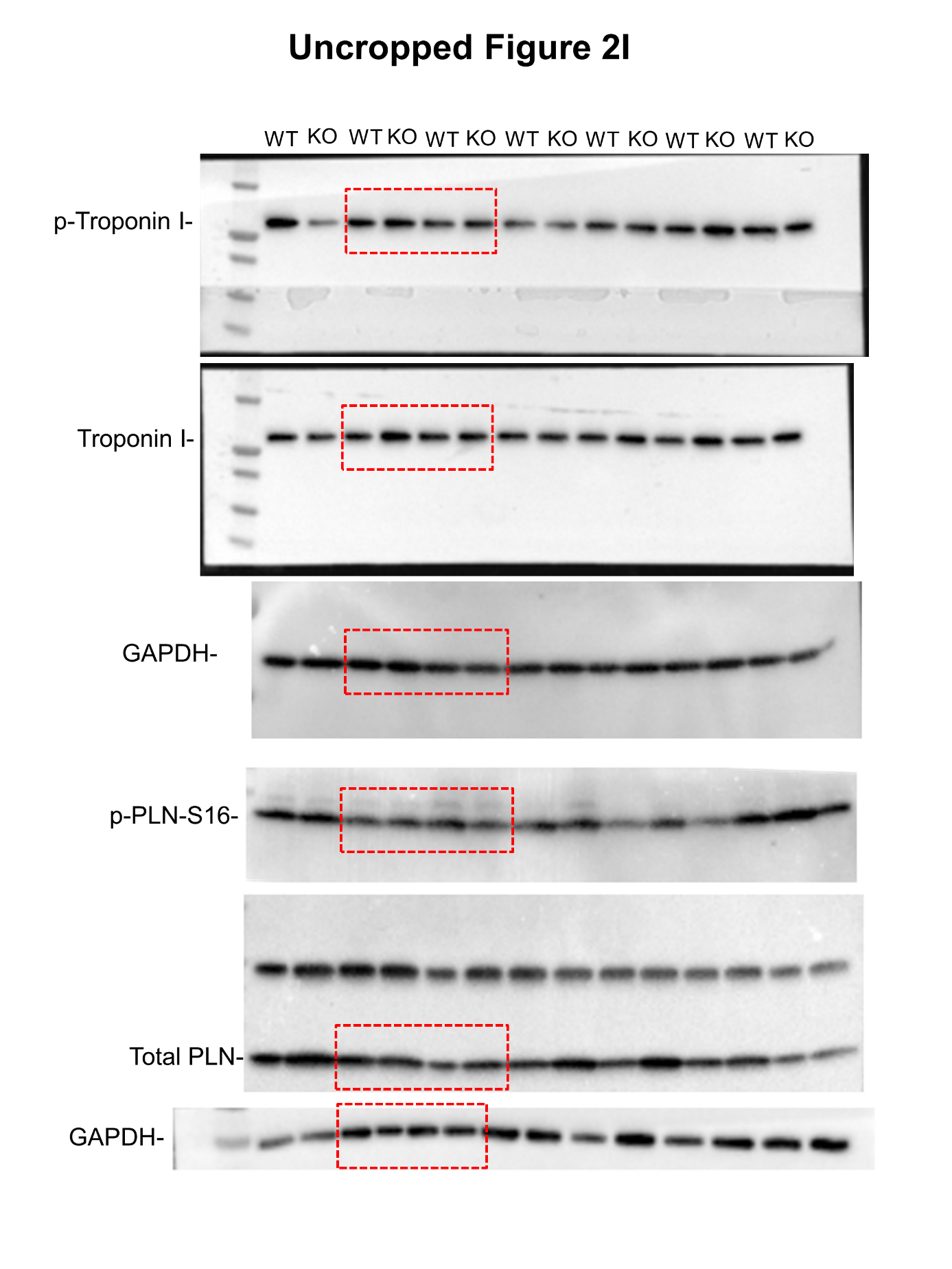


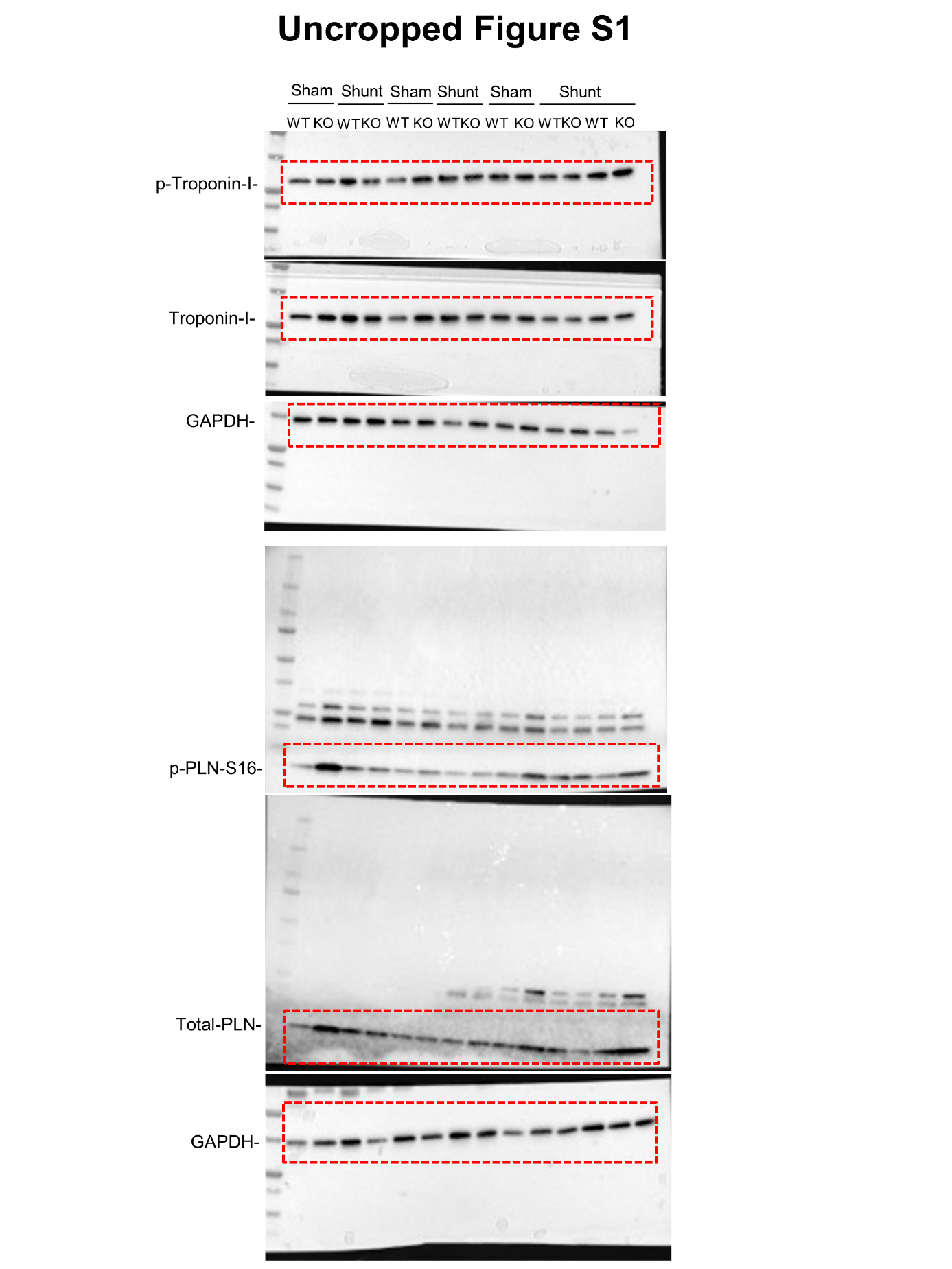

Supplement: Supplementary file 1 — Appendix S1 [file JCMM-26-4440-s001.docx]
